# Encoding of movement in near extrapersonal space in primate area VIP

**DOI:** 10.3389/fnbeh.2013.00008

**Published:** 2013-02-13

**Authors:** Frank Bremmer, Anja Schlack, André Kaminiarz, Klaus-Peter Hoffmann

**Affiliations:** ^1^Department of Neurophysics, Philipps-Universität MarburgMarburg, Germany; ^2^General Zoology and Neurobiology, Ruhr Universität BochumBochum, Germany

**Keywords:** parietal cortex, macaque monkey, VIP, self-motion, disparity, multisensory

## Abstract

Many neurons in the macaque ventral intraparietal area (VIP) are multimodal, i.e., they respond not only to visual but also to tactile, auditory and vestibular stimulation. Anatomical studies have shown distinct projections between area VIP and a region of premotor cortex controlling head movements. A specific function of area VIP could be to guide movements in order to *head for* and/or to *avoid* objects in near extrapersonal space. This behavioral role would require a consistent representation of visual motion within 3-D space and enhanced activity for nearby motion signals. Accordingly, in our present study we investigated whether neurons in area VIP are sensitive to moving visual stimuli containing depth signals from horizontal disparity. We recorded single unit activity from area VIP of two awake behaving monkeys (*Macaca mulatta*) fixating a central target on a projection screen. Sensitivity of neurons to horizontal disparity was assessed by presenting large field moving images (random dot fields) stereoscopically to the two eyes by means of LCD shutter goggles synchronized with the stimulus computer. During an individual trial, stimuli had one of seven different disparity values ranging from 3° uncrossed- (far) to 3° crossed- (near) disparity in 1° steps. Stimuli moved at constant speed in all simulated depth planes. Different disparity values were presented across trials in pseudo-randomized order. Sixty-one percent of the motion sensitive cells had a statistically significant selectivity for the horizontal disparity of the stimulus (*p* < 0.05, distribution free ANOVA). Seventy-five percent of them preferred crossed-disparity values, i.e., moving stimuli in near space, with the highest mean activity for the nearest stimulus. At the population level, preferred direction of visual stimulus motion was not affected by horizontal disparity. Thus, our findings are in agreement with the behavioral role of area VIP in the representation of movement in near extrapersonal space.

## Introduction

Self-motion through an environment induces a variety of sensory signals: visual, somatosensory, auditory, and vestibular. From a theoretical point of view, neurons responding to two or more sensory modalities appear well suited to unequivocally encode self-motion. The macaque ventral intraparietal area (area VIP) has been shown to respond to all four sensory modalities (Colby et al., 1993; Duhamel et al., 1998; Bremmer et al., 2002a,b; Schlack et al., 2002, 2005; Bremmer, 2005). Remarkably, individual neurons' receptive fields as determined by visual and auditory as well as visual and tactile responses have been shown to be spatially congruent (Avillac et al., 2005; Schlack et al., 2005). Such neurons allow to encode the spatial position of an external stimulus in a supramodal frame of reference.

The visual modality is the dominating sense in area VIP as most of the neurons respond to visual stimuli (Colby et al., 1993; Duhamel et al., 1998; Bremmer et al., 2002b; Schlack et al., 2002). Many of such neurons respond to real self-motion as well as to visual stimuli mimicking self-motion (Zhang and Britten, 2004; Zhang et al., 2004; Britten, 2008; Bremmer, 2011; Chen et al., 2011). Different from area MST, however, the preferred directions of self-motion as implied by the individual (visual, rotational vestibular, and tactile) directional preferences are always synergistically organized in area VIP (Bremmer et al., 2002b). This response property previously has been termed *action congruency*.

While vision and audition are so-called far senses, somatosensation is a near-sense. The question arises, whether or not response properties like preferred directions of motion are congruent across these different parts in space. Earlier as well as most recent studies had shown that many neurons in area VIP respond preferentially to stimuli presented close to the monkey. Colby and colleagues were the first to show responses of VIP neurons to monocularly presented ultra-near stimuli (Colby et al., 1993). Bremmer and Kubischik presented preliminary data showing by means of stereoscopic presentation a preference of VIP neurons to respond to stimuli presented in near as compared to far space (Bremmer and Kubischik, 1999). These latter findings were recently confirmed by Yang et al. (2011).

Based on the above described response properties it was suggested that area VIP might be highly relevant for the encoding of motion and/or movement in near extrapersonal space (Bremmer et al., 2002b). In everyday life this functional property would be extremely useful for the guidance of head-movements toward targets in a dense environment. In such case, near visual stimuli such as leaves of trees become tactile stimuli as the animal moves forward. As an alternative explanation it was suggested that area VIP might be critical for the avoidance of obstacles or fast approaching objects (Cooke et al., 2003; Graziano and Cooke, 2006a). This hypothesis was based on the finding that prolonged stimulation of macaque area VIP induces aversive hand and head movements. In both cases, i.e., the guidance of head-movements in order to *approach* targets or to *avoid* them, it would be of critical importance that the directional tuning for motion would be constant across near and far extrapersonal space. In addition, if VIP would have an alerting function, as would be expected in the case of an avoidance (re-)action, the average response of VIP neurons for near stimuli should be higher than the one for far stimuli.

Accordingly, in our present study we tested for these functional hypotheses with high behavioral relevance, i.e., (1) a stronger response for near as compared to far stimuli as well as (2) consistency of preferred direction of visual motion across near and far space.

## Materials and methods

We recorded neuronal activity in area VIP of two male awake behaving monkeys (*Macaca mulatta*: 9.2 kg and 9.5 kg). All treatments of the animals such as housing and surgical procedures were in accordance with German and international published guidelines on the use of animals in research (European Communities Council Directive 86/609/ECC).

### Animal preparation and experimental equipment

Before the experimental period, the animal had a head holding device implanted under general anesthesia. For the monitoring of eye movements, two scleral search coils were implanted. On the basis of previously measured MRI scans, we placed the recording chamber for microelectrode penetrations with its long axis parallel to the intraparietal sulcus and orthogonal to the scull. In one animal we recorded from the left, in the other animal from the right cortical hemisphere.

During experiments, monkeys were seated in a primate chair with the head fixed. For each correct trial monkeys received a liquid reward. We used a PC running self-written software (NABEDA, developed by Dr. M. Pekel) to control the stimulation and data acquisition. For each penetration we determined the location of area VIP by the position and depth of the electrode and by physiological criteria (e.g., direction selective responses to visual stimuli).

### Visual stimulation

**Figure 1:**
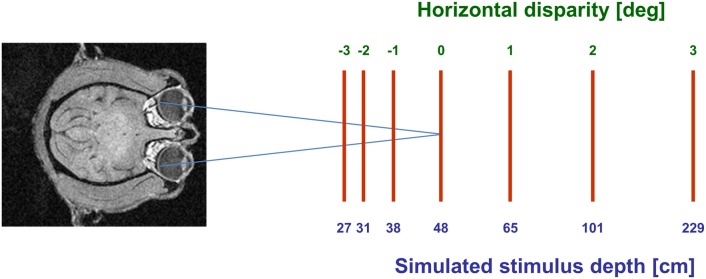
**Graphical scheme of the experimental setup.** Monkeys always fixated a target on the screen (blue axes). Moving stimuli were presented at seven different disparities, ranging from 3° crossed disparity (near space; negative values) to 3° uncrossed disparity (far space; positive values). These disparity values corresponded to simulated depths ranging from 27 cm to 229 cm in front of the monkey.

Visual stimuli (CRT video beamer, Elektrohome 4100) and the fixation target (power LED) were back-projected onto a screen subtending the central 70° by 70° of the visual field. During visual stimulation the monkey fixated a central target at screen distance (48 cm). The stimuli were random dot patterns (RDPs, i.e., 441 white dots on a black background) covering the full projection area. Stimuli were generated by distributing dot-stimuli by a pseudo-random process in a 2-D frontal plane, i.e., they did not contain motion-parallax cues. RDPs moved on a circular pathway thereby simulating self-motion in the frontoparallel plane. This approach allows to present all possible motion directions in a frontoparallel plane within a single trial (Schoppmann and Hoffmann, 1976; Bremmer et al., 2010). Stimuli moved at constant speed in all simulated depth planes. Sensitivity of neurons to horizontal disparity was assessed by presenting the RDPs stereoscopically to the two eyes by means of LCD shutter goggles (Silicon Graphics, customized) synchronized with the stimulus computer (Silicon Graphics, Indigo). Stimuli were presented at 120 Hz interlaced, i.e., at 60 Hz to each eye. During an individual trial, stimuli had one of seven different disparity values ranging from 3° uncrossed disparity (far space; positive values) to 3° crossed disparity (near space; negative values) in 1° steps. These disparity values corresponded to simulated depths ranging from 27 cm to 229 cm in front of the monkey (see Figure 1). Different disparity values were presented across trials in pseudo-randomized order.

### Data analysis

In a first step of our analysis we determined whether cells had a significant stimulus driven response. To this end, we selected for each neuron individually a response windows around the neuron's preferred direction and compared the discharges across the different trials for each disparity as well as for baseline activity by means of a distribution-free analysis of variance (ANOVA). These cells were considered for further analysis and we determined the subgroup with a significant tuning to horizontal disparity. To this end, an ANOVA was applied to the same discharges, yet, without baseline activity. For those remaining cells we determined, cell by cell, for all stimulus disparities whether or not responses were significantly tuned for the direction of visual stimulus motion (Rayleigh's test). This final test allowed us to determine whether direction tuning stayed constant or rather changed systematically across space in front of the monkey by pairwise comparison of the preferred direction obtained for the different depth planes.

## Results

**Figure 2:**
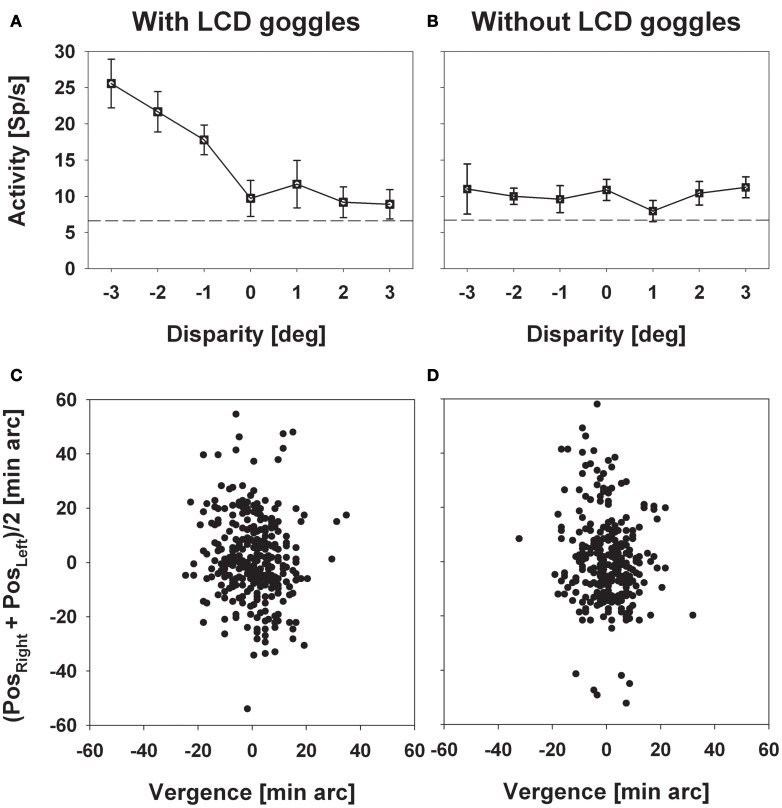
**Experimental validation of the stereoscopic setup.** The left and the right column depict data obtained with (left) and without (right) the LCD goggles being synchronized with the stimulus computer. Panels in the upper row (**A** and **B**) show mean discharges (±standard error) of neural activity for stimuli presented at a certain disparity (depth, only perceivable with the goggles being synchronized). Panels in the bottom row (**C** and **D**) show the monkey's oculomotor behavior during a sample trial each. In these panels, the ordinate depicts the mean eye position of both eyes while the abscissa depicts vergence. Neural activity strictly depended on horizontal disparity, while oculomotor behavior, which was perfectly centered on zero vergence, did not.

We recorded from 320 cells in two hemispheres of two macaque monkeys. Two hundred and thirty (72%) responded significantly (distribution free ANOVA, *p* < 0.05) to visual stimulus motion as induced by a circular pathway stimulus thereby confirming results from previous studies (Bremmer et al., 2002a). The circular pathway stimulus allows to present all possible stimulus directions (SDs) within a frontoparallel plane within a single trial (see section “Materials and Methods”). Across trials, stimuli were presented at constant speed in pseudo-randomized order in different depth planes in front of the monkey by means of LCD shutter goggles which were synchronized with the stimulus computer. The appropriateness of this approach was confirmed in a number of cells by comparing stimulus responses with and without goggles. An example for such a comparison is shown in Figure 2. Panel (**A**) shows the responses (±standard error) for stimuli moving into a neuron's preferred direction as determined by the circular pathway stimulus. For this neuron the strongest discharge was obtained by the stimulus moving in the frontoparallel plane being closest to the monkey (ANOVA, *p* < 0.001). Response strength decreased continuously for stimuli being presented further away and remained slightly above spontaneous discharge for stimuli presented in the plane of fixation and beyond. With the goggles removed stimuli no longer contained horizontal disparity information, because stimuli no longer were presented exclusively to the one eye or the other, but rather were presented to both eyes. As expected, responses did not differ for the different stimuli (*p* > 0.4) (Figure 2B). The quality of fixation did not differ between the two cases, i.e., with and without goggles (Figures 2C,D).

### Response strength

**Figure 3:**
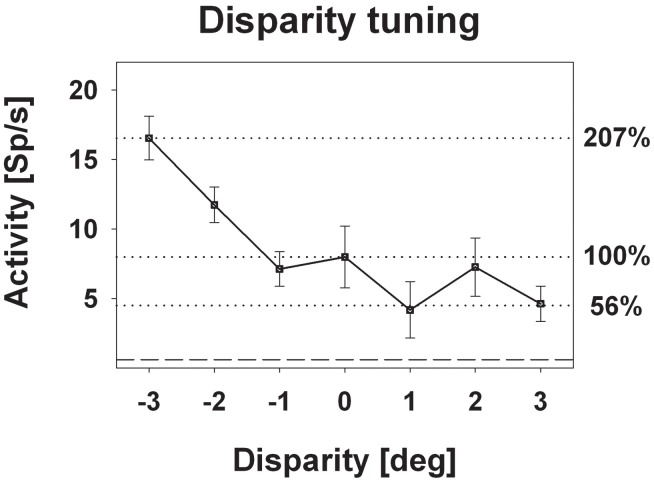
**Disparity tuning: single cell level.** The diagram shows the disparity tuning of a representative neuron (mean discharge ±standard error). The horizontal dotted lines indicate the mean discharge for stimuli presented at screen distance, i.e., zero disparity, and those disparities evoking the maximum (−3° disparity) and the minimum (+1° disparity) response. Activity induced by stimuli at zero disparity was normalized to 100%.

**Figure 4:**
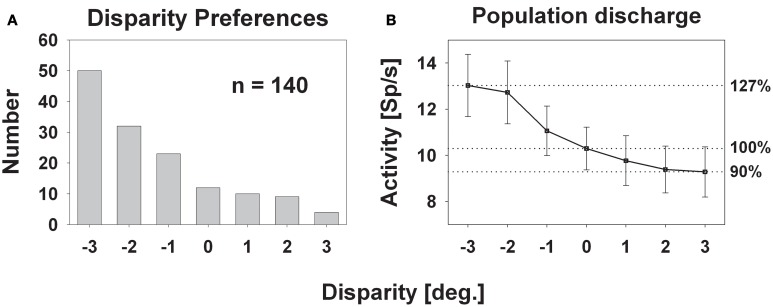
**Disparity tuning: population level.** The histogram in panel **(A)** shows the number of cases for which the peak discharge of a single cell was found at a certain disparity. The largest number (50) was found for the stimulus closest to the monkey: −3° disparity corresponding to 27 cm in front of the monkey. The smallest number (4) occurred for the stimulus furthest away, i.e., at +3° disparity, corresponding to 229 cm in front of the monkey. Panel **(B)** depicts the mean population activity (±standard error) taken from all 140 neurons with a significant response to visual stimulus motion and a significant tuning for horizontal disparity.

Response strength was significantly tuned to horizontal disparity in 140 cells (61%, distribution free ANOVA, *p* < 0.05). Tuning was mostly broad, as shown in Figure 3. Like in the previous example, peak discharge was observed for the stimulus closest to the monkey, i.e., at −3° disparity, corresponding to a stimulus plane 27 cm in front of the monkey. For this cell, response strength didn't stay constant for stimuli beyond the plane of fixation but further decreased. Both response features, peak discharge for stimuli closest to the monkey and decreasing response strength for stimuli beyond the plane of fixation, were also observed at the population level (Figure 4). Panel (**A**) depicts the distribution of response peaks of individual cells at a certain distance (or disparity) relative to the monkey. For 50 cells (36%) the peak response was observed for stimuli 27 cm in front of the monkey, corresponding to −3° disparity. A total of 105 cells (75%) preferred stimuli in near space while, i.e., these cells had their peak discharge with one of the stimuli presented at crossed (negative) disparity. Only 23 cells (16%) preferred stimulus motion in far space, i.e., had their peak discharge with one of the uncrossed (positive) disparities beyond the plane of fixation. Like in the single cell examples the population's average response increased in near space and was strongest for the nearest stimulus plane (Panel **B**). When normalizing the response in the plane of fixation to a value of 100%, the response increased to 127% for the closest stimuli. It decreased, on the other hand, continuously for stimuli further away with the weakest response (90%) obtained for stimuli at 3° uncrossed disparity corresponding to a distance of 229 cm in front of the monkey.

**Figure 5:**
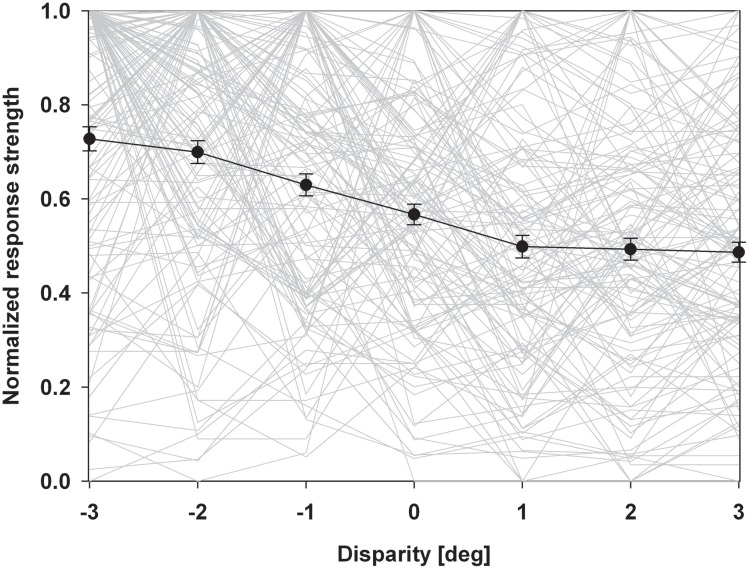
**Normalized population discharge.** In this population analysis, we first normalized maximum discharges of each neuron to a value of 1.0. The resulting disparity tuning curves of each neuron are shown as light grey lines. We then averaged these normalized response curves (solid black line). Error bars indicate the standard error. The resulting response curve was strongly monotonic with the strongest discharge for the closest stimulus.

An alternative form of population analysis is shown in Figure 5. Here, we first normalized all responses to a value of 1. This approach has the advantage that tuning shapes can be compared independent of firing rates. The average normalized response curve was strongly monotonic with the highest response at the closest stimulus. A distribution free ANOVA indicated that differences of the normalized discharges were highly significant (*p* < 0.001). A pairwise comparison indicated that all values were significantly different from each other, except those for the stimuli beyond the plane of fixation.

### Directional selectivity: single cell level

**Figure 6:**
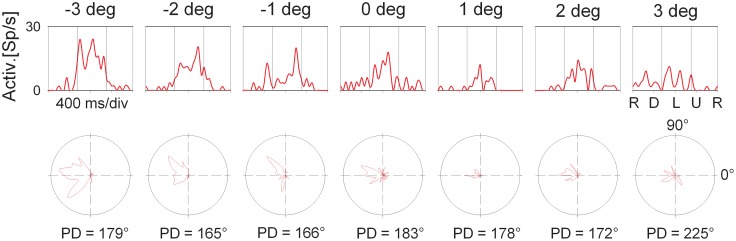
**Directional tuning as a function of disparity: single cell level.** The panels in the top row shows the response (spike density function) to a complete cycle (corresponding to 1600 ms) of the circular pathway stimulus of the neuron whose average discharge for the preferred direction is shown in Figure 3. The sequence of stimulus directions is shown on the very right: right (R), down (D), left (L), up (U), and again right. The vertical lines indicate the interval which was considered to compute this average discharge. The bottom row shows the same data in polar plots. The preferred directions (PD) are computed based on vector averaging, i.e., weighting each motion direction with the neuron's discharge for this direction.

The employment of the circular pathway stimulus allowed to map the complete tuning of individual cells by presenting all possible SDs in the frontoparallel plane. This approach provides the same results as conventional testing with linear motion into different (typically eight) directions. It has the advantage, however, to be much faster than the conventional testing because all SDs are presented within a single trial (for details, see Bremmer et al., 2002a). Figure 6 shows the directional response of the cell whose disparity tuning had been depicted in Figure 3. The panels in the upper row show the cell's response to the circular pathway stimulus presented at the different disparity values. The panels in the bottom row show the same data in polar plots. The vertical lines in the top panels demarcate the part of the response which was selected for determining the cell's response strength. Preferred directions as indicated below each polar plot were determined by means of vector averaging. For this neuron, all directional tunings were significant as determined by Rayleigh's test (*p* < 0.05). Directional preference was not identical across space. Instead, it varied between *PD* = 165° (obtained at 2° crossed disparity) and *PD* = 225° (obtained for stimuli present at 3° uncrossed disparity). The directional preference at screen distance (zero disparity) was in the middle range (*PD* = 183°).

In order to quantify the consistency of the directional tuning of cells across space we computed on a pairwise basis the difference between the preferred directions at the various disparities, yet, only for those pairs for which both directional tunings were significant. Rayleigh's test confirmed significant tuning for all disparity values for the responses shown in Figure 6, resulting in a total of 21 pairwise comparisons (*PD*_smaller disparity_−*PD*_larger disparity_) as listed in Table 1.

**Table 1:** **Pairwise differences between preferred directions of moving visual stimuli presented at different horizontal disparities**.

**Diaparity [°]/PD [°]**	**−3/179**	**−2/165**	**−1/166**	**0/183**	**1/178**	**2/172**	**3/225**
−3/179		14	13	−4	1	7	−46
−2/165			−1	−18	−13	−7	−60
−1/166				−17	−12	−6	−59
0/183					5	11	−43
1/178						6	−47
2/172							−53
3/225							

As a difference between (*PD*_A_−*PD*_B_) equals the negative of (*PD*_B_−*PD*_A_), only half of the entries of this matrix table are filled. For this neuron, differences in preferred direction varied between +14° (*PD*_−3°_−*PD*_−2°_) and −60° (*PD*_−2°_−*PD*_3°_), resulting in an average angular difference of $\bar{\left(PDn^{{}^{\circ}}-PDm^{{}^{\circ}}\right)}=-15.7^{{}^{\circ}}$.

### Directional selectivity: population level

**Figure 7:**
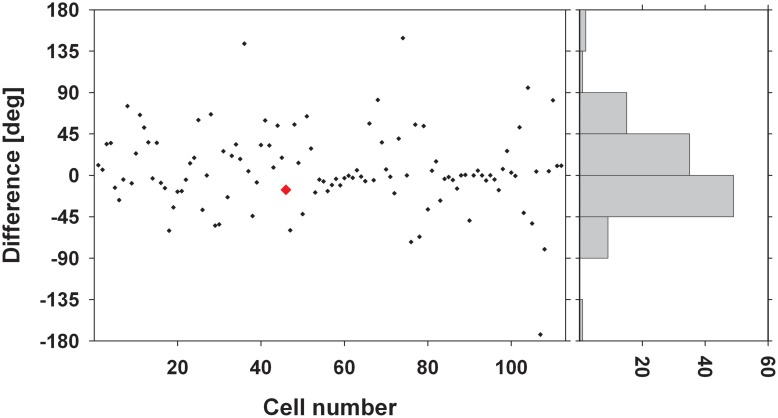
**Average angular differences in directional tuning.** The scatter plot shows for each neuron the average angular difference between preferred directions (PDs) of visual motion at different disparities. The red symbol indicates the value from the neuron whose disparity tuning was shown in Figures 3 and 6. The histogram on the right gives the distribution of these angular differences sorted in 45° bins. Seventy-five percent of the cells showed a mean angular difference of less than ±45°.

**Figure 8:**
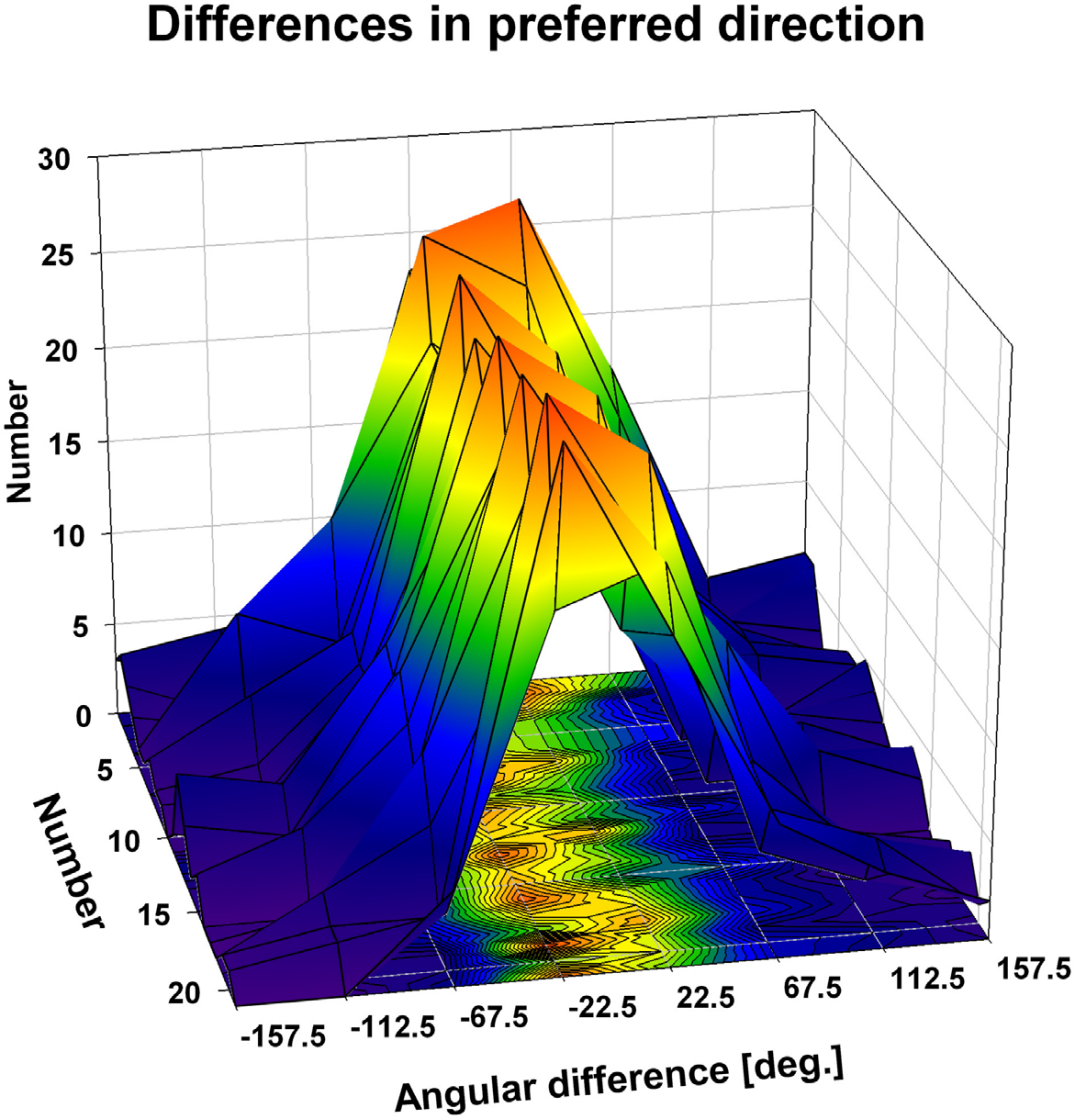
**Directional tuning as a function of disparity: population level.** The three-dimensional plot shows color coded the distribution of the angular differences between preferred directions (PDs) of visual motion at different disparities. We computed for each neuron on a pairwise basis the angular difference between PDs obtained at different disparities in case the tunings were significant (Rayleigh test, *p* < 0.05). Given the seven different disparity values, this resulted in a maximum of 21 pairwise comparisons per neuron, represented by the *y*-axis of the *x*-*y*-base of the cube. Values were sorted for each condition (1–21) into 45° bins (*x*-axis of the *x*-*y*-base). For all conditions, the angular differences clustered in the small value bins, i.e., around zero.

**Figure 9:**
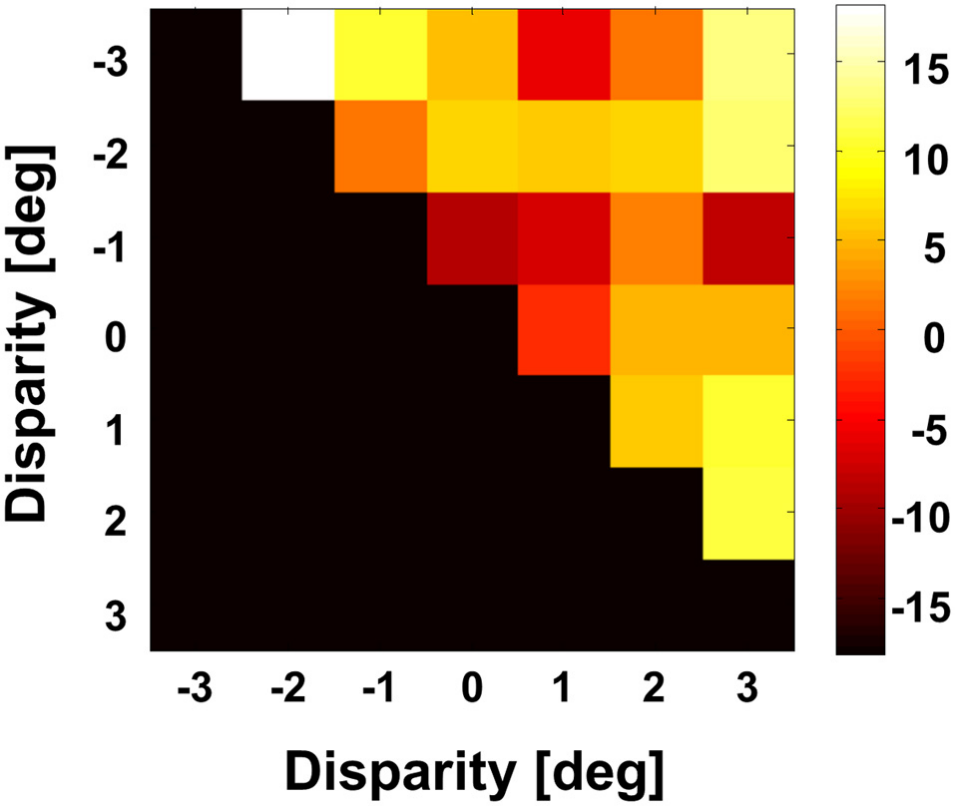
**Angular differences between preferred directions obtained at different disparities.** We computed for each neuron on a pairwise basis the angular difference between PDs obtained at different disparities in case the tunings were significant (Rayleigh test, *p* < 0.05). For this analysis, we averaged for each pairwise comparison the angular difference. The grand average as computed for the full population is shown color coded. None of the values was different from zero (Bonferroni correction, *t*-test, *p* > 0.3).

We were interested in the stability of the directional tuning for visual motion across space in area VIP as a whole. To this end, we performed two different population analyses, both being based on data from neurons with significant disparity tuning and responses with a significant directional tuning obtained at one or more disparity values. In a first analysis, we considered only those disparity-tuned neurons which had a significant directional tuning at a minimum of two different disparities each (*n* = 112). This allowed to compute on a cell-by-cell basis the average of the pairwise differences in direction tuning as in the example above. For 75% of the neurons (84/112) the average angular difference of directional tuning obtained at two different disparities was smaller than ±45° (Figure 7). For the population of neurons (*n* = 112) the mean of these average angular differences was 5.7°, which was statistically not different from zero (one-sided *t*-test, *p* > 0.15). In a second analysis, we determined the angular differences in directional tuning individually for each of the 21 pairs of disparity values, resulting in a total of 1397 pairwise comparisons. For each disparity pair, responses from 53 to 85 neurons contributed to the analysis. Data were sorted in 45°-bins, the distribution of which is shown in Figure 8. For all 21 disparity pairs, angular differences centered around small values. A different graphical depiction of this second analysis is shown in Figure 9. Each pixel in this matrix indicates the average value of angular differences between discharges obtained for a certain pair of disparities. As can be estimated from the color code, the population values clustered around zero. A Bonferroni-corrected one-sided *t*-test revealed for none of the pixels a significant deviation from zero (*p* > 0.05). Both population analyses therefore unequivocally showed that the directional tuning of area VIP neurons is constant within the space in front of the monkey.

## Discussion

In our study we have investigated the tuning for horizontal disparity in macaque area VIP. The vast majority of cells were tuned for near space, with more than a third of the cells having their response maximum at the nearest stimulus. The average response of our population of cells was strongest for the stimulus closest to the monkey. Stimuli beyond the plane of fixation evoked responses which decreased with increasing distance. Directional tuning was constant across the space in front of the monkey. In the following, we will discuss the relationship of these functional properties with the suggested behavioral role of area VIP in primates.

### The subdivisions of near space

The vast majority of cells responded most strongly for stimuli being closer to the monkey than the plane of fixation. Accordingly, these latter stimuli were within the so-called *peripersonal* space, i.e., within the monkey's reach distance. Hence, while stimuli were presented along a continuum of disparities, the simulated stimulus distances moved from one part of space (*near-extrapersonal*) into a different part of space (*peripersonal*). Previous studies had shown that neurons in area VIP respond strongly to ultra-near stimuli (Colby et al., 1993). This leads to the question whether the disparity tuning bias for near disparities as observed in our current study also would have been present if stimuli had been presented exclusively within peripersonal space. If neurons were pure *peripersonal-space-encoders*, they might not reveal a disparity tuning within this part of space. As shown in Figures 4 and 5, response strength as well as the number of response peaks increased for stimuli with decreasing distance from the monkey within peripersonal space. Therefore, these data are clear evidence for a disparity tuning within peripersonal space. Yet, the differences decreased suggesting a saturation of responses (i.e., a loss of disparity tuning) for stimuli being even closer to the animal. Hence, area VIP might show disparity tuning only in the outer part of peripersonal space. Further experiments are needed, however, to test for this hypothesis.

### Response strength

The vast majority of cells responded more strongly to stimuli with crossed disparity, i.e., stimuli closer to the monkey than the plane of fixation. This finding is in line with previous results from Colby et al. (1993) and our own group (Bremmer and Kubischik, 1999) and has recently been confirmed by Yang et al. (2011). A majority of cells with response peaks for stimuli in near space does not necessarily result in a stronger population discharge for near stimuli. Theoretically, few neurons (16% in our case) having their response maximum beyond the plane of fixation, could fire at high rate thereby (over-)compensating the population activity of a larger number of neurons (75% in our case) firing at lower rate for stimuli in near space. This, however, was not the case in our study. Instead, when normalizing the discharge related to stimuli presented in the plane of fixation to a value of 100%, neurons responded on average at 127% to stimuli presented closest to the monkey, i.e., 27 cm in front of the monkey. The question arises, whether or not a response change by approximately 25% is of critical relevance for the processing of visual stimuli. From our point of view, this is indeed the case. Evidence for this understanding comes from experiments employing completely different behavioral tasks. One such example are experiments by Treue and Maunsell (1999) who required monkeys to shift their attention to certain stimulus features while recording in areas MT and/or MST in the superior temporal sulcus. The response increase due to a shift of attention was approximately 25%. Some of the neurons in our sample revealed even a much stronger effect of enhancement for nearby stimuli as shown for an example cell in Figure 3. The responses of this neuron increased to 207% with respect to the discharges found at zero disparity. This increase by more than 100% is even stronger than the increase reported by Cook and Maunsell (2002), who reported a median increase in area VIP by some 60% when animals were involved in an attentional task. While the experimental paradigms were completely different in our current study and in the attentional experiments, the observed effects of horizontal disparity on neuronal discharge had the same order of magnitude. Along the same vein, we considered the reduction of the population activity as induced by stimuli beyond the plane of fixation, which was in the order of 10%. A similar decrease in stimulus driven responses was reported by Bremmer and colleagues when presenting visual stimuli around the time of saccades (Bremmer et al., 2009). Visual stimuli typically go unnoticed during saccades. In other words, a reduction of neural activity by some 10% might point toward a strong perceptual attenuation of visual stimuli beyond the plane of fixation. In summary, these different behavioral scenarios are accompanied by similar changes in neural activity. This clearly indicates that neurons are active in a multi-dimensional parameter space and neural activity can be influenced (enhanced or suppressed) similarly although the underlying behaviors are critically different.

The increasing response to stimuli with decreasing distance to the monkey can mathematically be described as a gradient. Our finding of a strong gradient leads to the question about its behavioral relevance. It might be a hint toward an alerting system for near and ultra-near stimuli (Colby et al., 1993). Along this vein, Graziano and colleagues were able to show that prolonged microstimulation in area VIP induces complex, defensive movements (Cooke et al., 2003). The authors suggested that VIP contributes to defensive movements triggered by stimuli on or near the head. This behavior resembles the one found after manipulation of activity in the so-called polysensory zone in frontal cortex (Cooke and Graziano, 2004a,b). Both areas are interconnected and are discussed to be a critical part of a network of areas relevant for defensive behavior (Graziano and Cooke, 2006b). Our data are in line with this behavioral hypothesis.

### Directional preference in 3D space

As an alternative to the above described defensive behavior it has been suggested that area VIP might be critically involved in the active guidance of self-motion in near space. This hypothesis was deduced from the finding that many neurons in area VIP responded to visual and rotatory vestibular stimulation and that in such case preferred directions were co-aligned (Bremmer et al., 2002b). In addition, many visual and tactile receptive fields of single VIP neurons are spatially congruent (Duhamel et al., 1998; Avillac et al., 2005). Both observations are complemented by the finding that responses are “action-congruent,” i.e., a preference for tactile motion to the right or vestibular preferred direction to the right predicts a preference for rightward visual motion when tested with a stimulus at viewing distance with high reliability (Bremmer et al., 2002b). Accordingly, the hypothesis of a behavioral role of area VIP in the active guidance of self-motion appears plausible considering self-motion in a dense, natural environment. In such case, visual stimuli become at some point tactile stimuli when the animal moves forward. If such neurons were multimodal, as is often the case in area VIP (Bremmer, 2005, 2011; Schlack et al., 2005), they could encode the self-motion direction in a supramodal frame of reference, i.e., regardless of whether they were driven by visual, tactile, auditory, or vestibular stimulation.

Such a consistent encoding, however, would require a consistent directional preference for visual motion within full 3-D space. Previous studies had provided first evidence for such a consistency (Bremmer and Kubischik, 1999; Yang et al., 2011). The authors of the latter study had compared the relationship between responses to visually simulated and real self-motion in the dorsal aspect of the medio-superior-temporal area (area MSTd) and area VIP of the macaque. Both areas are at the same hierarchical level of the visual processing stream (Hilgetag et al., 1996) and neurons in both areas respond to real or simulated translational self-motion (Duffy, 1998; Bremmer et al., 1999, 2002b; Schlack et al., 2002; Gu et al., 2006, 2008, 2010; Chen et al., 2011). The comparison of the response properties was based on two previous findings: (1) for many neurons in both areas, preferred directions for real and visually simulated translational self-motion are not congruent but rather opposite; (2) about 40% of the neurons in area MSTd had been reported to reverse their disparity preference for opposite directions of motion (Roy and Wurtz, 1990). Based on these findings, it had been suggested that such neurons may play an important role in (1) dissociating self- from object-motion and in (2) representing self-motion during fixation on a world-fixed target (Roy et al., 1992). In order to verify or falsify this hypothesis, Yang and colleagues investigated for eight frontoparallel SDs the relationship between neurons' disparity sensitivity and self-motion preference. For area MSTd the authors could show that disparity preference can reverse for opposite motion directions, while direction preference for visual motion must not necessarily reverse for near vs. far disparities. In their study, about one fourth [compared to 40% in the study of Roy and Wurtz (1990)] of the disparity tuned MSTd cells revealed a direction-dependent disparity tuning (DDD). Only very few VIP neurons (5 out of approximately 100 neurons under investigation) showed such a response behavior. Most interestingly, in both areas an opposite preference for visual and vestibular self-motion was not linked to a DDD behavior concerning horizontal disparity. In our current study we tested not only eight stimulus directions, i.e., four directions and four opposite directions. Instead, the use of the circular pathway stimulus allowed to map continuously all possible directions in frontoparallel space. Our data unequivocally show that preferred stimulus motion direction is constant across full 3-D-space in front of the monkey.

### Parietal cortex and near space

Our data are in line with two hypotheses concerning the behavioral role of macaque area VIP: (1) the control of defensive movements concerning approaching objects and (2) the guidance of (head) movements in a dense environment. It seems likely that area VIP subserves both functions since from a behavioral point of view they do not exclude each other. Area VIP is not the only parietal area showing an overrepresentation of near space. Genovesio and Ferraina (2004) investigated depth tuning in the neighboring lateral intraparietal area (area LIP) of the macaque. The saccade tuning of LIP cells was modulated by fixation distance: average population activity increased for closer fixation. The authors concluded that the discharges of a population of LIP neurons are suited to encode the position of objects in 3-D space. Similar findings have been reported for the parietal reach region (PRR) by Bhattacharyya et al. (2009). These authors found that individual PRR neurons encode depth *relative* to the fixation point, i.e., in eye centered coordinates. The activity of individual neurons was modulated by vergence angle. The authors concluded that *absolute* depth could be decoded from a population of PRR neurons. A clear overrepresentation of peripersonal as compared to near extrapersonal space has also been found in area V6A in the parieto-occipital sulcus (Hadjidimitrakis et al., 2011, 2012; Breveglieri et al., 2012). In these studies, monkeys had to saccade to or fixate targets at different eccentricities (version) and depths (vergence). Many neurons were modulated by both parameters. Population activity was significantly stronger for targets in peripersonal space. Within this reach space, however, neurons did not have any depth preference.

The above described findings in the rhesus monkey apply also to human parietal cortex. Quinlan and Culham (2007) have shown a strong overrepresentation of peripersonal space in the dorsal parieto-occipital sulcus (dPOS). Subjects viewed moving (looming and receding) visual stimuli at three distances: near, medium, and far, in the fMRI scanner. BOLD contrast increased for closer stimuli. Preliminary data from our own lab provide evidence for a massive overrepresentation of near space in human posterior parietal cortex. We presented in the fMRI scanner visual stimuli moving in frontoparallel planes either in near or in far space, while subjects fixated at constant screen distance. We found enhanced BOLD contrast in an area most likely being the human counterpart of macaque area VIP (Bremmer et al., 2001a,b). Accordingly, we suggest that area VIP plays a critical role in the encoding of motion in near extrapersonal space in primates, i.e., humans and monkeys.

### Conflict of interest statement

The authors declare that the research was conducted in the absence of any commercial or financial relationships that could be construed as a potential conflict of interest.

## References

[B1] Avillac M. Deneve S. Olivier E. Pouget A. Duhamel J. R. (2005). Reference frames for representing visual and tactile locations in parietal cortex. Nat. Neurosci. 8, 941–949 10.1038/nn1480 15951810

[B2] Bhattacharyya R. Musallam S. Andersen R. A. (2009). Parietal reach region encodes reach depth using retinal disparity and vergence angle signals. J. Neurophysiol. 102, 805–816 10.1152/jn.90359.2008 19439678 PMC2724352

[B3] Bremmer F. (2005). Navigation in space–the role of the macaque ventral intraparietal area. J. Physiol. 566, 29–35 10.1113/jphysiol.2005.082552 15845586 PMC1464721

[B4] Bremmer F. (2011). Multisensory space: from eye-movements to self-motion. J. Physiol. 589, 815–823 10.1113/jphysiol.2010.195537 20921203 PMC3060361

[B5] Bremmer F. Duhamel J.-R. Ben Hamed S. Graf W. (2002a). Heading encoding in the macaque ventral intraparietal area (VIP). Eur. J. Neurosci. 16, 1554–1568 10.1046/j.1460-9568.2002.02207.x 12405970

[B6] Bremmer F. Klam F. Duhamel J.-R. Ben Hamed S. Graf W. (2002b). Visual-vestibular interactive responses in the macaque ventral intraparietal area (VIP). Eur. J. Neurosci. 16, 1569–1586 10.1046/j.1460-9568.2002.02206.x 12405971

[B7] Bremmer F. Kubischik M. (1999). Representation of near extrapersonal space in the macaque ventral intraparietal area (VIP). Soc. Neurosci. Abstr. 471, 11 9425183

[B8] Bremmer F. Kubischik M. Hoffmann K. P. Krekelberg B. (2009). Neural dynamics of saccadic suppression. J. Neurosci. 29, 12374–12383 10.1523/JNEUROSCI.2908-09.2009 19812313 PMC2787621

[B9] Bremmer F. Kubischik M. Pekel M. Hoffmann K. P. Lappe M. (2010). Visual selectivity for heading in monkey area MST. Exp. Brain Res. 200, 51–60 10.1007/s00221-009-1990-3 19727690

[B10] Bremmer F. Kubischik M. Pekel M. Lappe M. Hoffmann K. P. (1999). Linear vestibular self-motion signals in monkey medial superior temporal area. Ann. N.Y. Acad. Sci. 871, 272–281 10.1111/j.1749-6632.1999.tb09191.x 10372078

[B11] Bremmer F. Schlack A. Hoffmann K.-P. Zilles K. Fink G. R. (2001a). Encoding of near space in the primate ventral intraparietal area. Soc. Neurosci. Abstr. 58, 1

[B12] Bremmer F. Schlack A. Shah N. J. Zafiris O. Kubischik M. Hoffmann K.-P. (2001b). Polymodal motion processing in posterior parietal and premotor cortex: a human fMRI study strongly implies equivalencies between humans and monkeys. Neuron 29, 287–296 10.1016/S0896-6273(01)00198-2 11182099

[B13] Breveglieri R. Hadjidimitrakis K. Bosco A. Sabatini S. P. Galletti C. Fattori P. (2012). Eye position encoding in three-dimensional space: integration of version and vergence signals in the medial posterior parietal cortex. J. Neurosci. 32, 159–169 10.1523/JNEUROSCI.4028-11.2012 22219279 PMC6621321

[B14] Britten K. H. (2008). Mechanisms of self-motion perception. Annu. Rev. Neurosci. 31, 389–410 10.1146/annurev.neuro.29.051605.112953 18558861

[B15] Chen A. DeAngelis G. C. Angelaki D. E. (2011). Representation of vestibular and visual cues to self-motion in ventral intraparietal cortex. J. Neurosci. 31, 12036–12052 10.1523/JNEUROSCI.0395-11.2011 21849564 PMC3169295

[B16] Colby C. L. Duhamel J. R. Goldberg M. E. (1993). Ventral intraparietal area of the macaque: anatomic location and visual response properties. J. Neurophysiol. 69, 902–914 8385201 10.1152/jn.1993.69.3.902

[B17] Cook E. P. Maunsell J. H. (2002). Attentional modulation of behavioral performance and neuronal responses in middle temporal and ventral intraparietal areas of macaque monkey. J. Neurosci. 22, 1994–2004 11880530 10.1523/JNEUROSCI.22-05-01994.2002 PMC6758868

[B18] Cooke D. F. Graziano M. S. (2004a). Sensorimotor integration in the precentral gyrus: polysensory neurons and defensive movements. J. Neurophysiol. 91, 1648–1660 10.1152/jn.00955.2003 14586035

[B19] Cooke D. F. Graziano M. S. (2004b). Super-flinchers and nerves of steel: defensive movements altered by chemical manipulation of a cortical motor area. Neuron 43, 585–593 10.1016/j.neuron.2004.07.029 15312656

[B20] Cooke D. F. Taylor C. S. Moore T. Graziano M. S. (2003). Complex movements evoked by microstimulation of the ventral intraparietal area. Proc. Natl. Acad. Sci. U.S.A. 100, 6163–6168 10.1073/pnas.1031751100 12719522 PMC156343

[B21] Duffy C. J. (1998). MST neurons respond to optic flow and translational movement. J. Neurophysiol. 80, 1816–1827 9772241 10.1152/jn.1998.80.4.1816

[B22] Duhamel J. R. Colby C. L. Goldberg M. E. (1998). Ventral intraparietal area of the macaque: congruent visual and somatic response properties. J. Neurophysiol. 79, 126–136 9425183 10.1152/jn.1998.79.1.126

[B23] Genovesio A. Ferraina S. (2004). Integration of retinal disparity and fixation-distance related signals toward an egocentric coding of distance in the posterior parietal cortex of primates. J. Neurophysiol. 91, 2670–2684 10.1152/jn.00712.2003 14960558

[B24] Graziano M. S. Cooke D. F. (2006a). Parieto-frontal interactions, personal space, and defensive behavior. Neuropsychologia 44, 845–859 10.1016/j.neuropsychologia.2005.09.009 16277998

[B25] Graziano M. S. Cooke D. F. (2006b). Parieto-frontal interactions, personal space, and defensive behavior. Neuropsychologia 44, 2621–2635 17128446 10.1016/j.neuropsychologia.2005.09.011

[B26] Gu Y. Angelaki D. E. DeAngelis G. C. (2008). Neural correlates of multisensory cue integration in macaque MSTd. Nat. Neurosci. 11, 1201–1210 10.1038/nn.2191 18776893 PMC2713666

[B27] Gu Y. Fetsch C. R. Adeyemo B. DeAngelis G. C. Angelaki D. E. (2010). Decoding of MSTd population activity accounts for variations in the precision of heading perception. Neuron 66, 596–609 10.1016/j.neuron.2010.04.026 20510863 PMC2889617

[B28] Gu Y. Watkins P. V. Angelaki D. E. DeAngelis G. C. (2006). Visual and nonvisual contributions to three-dimensional heading selectivity in the medial superior temporal area. J. Neurosci. 26, 73–85 10.1523/JNEUROSCI.2356-05.2006 16399674 PMC1538979

[B29] Hadjidimitrakis K. Breveglieri R. Bosco A. Fattori P. (2012). Three-dimensional eye position signals shape both peripersonal space and arm movement activity in the medial posterior parietal cortex. Front. Integr. Neurosci. 6:37 10.3389/fnint.2012.00037 22754511 PMC3385520

[B30] Hadjidimitrakis K. Breveglieri R. Placenti G. Bosco A. Sabatini S. P. Fattori P. (2011). Fix your eyes in the space you could reach: neurons in the macaque medial parietal cortex prefer gaze positions in peripersonal space. PLoS ONE 6:e23335 10.1371/journal.pone.0023335 21858075 PMC3157346

[B31] Hilgetag C. C. O'Neill M. A. Young M. P. (1996). Indeterminate organization of the visual system. Science 271, 776–777 10.1126/science.271.5250.776 8628990

[B32] Quinlan D. J. Culham J. C. (2007). fMRI reveals a preference for near viewing in the human parieto-occipital cortex. Neuroimage 36, 167–187 10.1016/j.neuroimage.2007.02.029 17398117

[B33] Roy J.-P. Komatsu H. Wurtz R. H. (1992). Disparity sensitivity of neurons in monkey extrastriate area MST. J. Neurosci. 12, 2478–2492 1613542 10.1523/JNEUROSCI.12-07-02478.1992 PMC6575856

[B34] Roy J.-P. Wurtz R. H. (1990). The role of disparity-sensitive cortical neurons in signalling the direction of self-motion. Nature 348, 160–162 10.1038/348160a0 2234078

[B35] Schlack A. Hoffmann K. P. Bremmer F. (2002). Interaction of linear vestibular and visual stimulation in the macaque ventral intraparietal area (VIP). Eur. J. Neurosci. 16, 1877–1886 10.1046/j.1460-9568.2002.02251.x 12453051

[B36] Schlack A. Sterbing-D'Angelo S. J. Hartung K. Hoffmann K. P. Bremmer F. (2005). Multisensory space representations in the macaque ventral intraparietal area. J. Neurosci. 25, 4616–4625 10.1523/JNEUROSCI.0455-05.2005 15872109 PMC6725030

[B37] Schoppmann A. Hoffmann K.-P. (1976). Continuous mapping of direction selectivity in the cat's visual cortex. Neurosci. Lett. 2, 177–181 10.1016/0304-3940(76)90011-2 19604837

[B38] Treue S. Maunsell J. H. (1999). Effects of attention on the processing of motion in macaque middle temporal and medial superior temporal visual cortical areas. J. Neurosci. 19, 7591–7602 10460265 10.1523/JNEUROSCI.19-17-07591.1999 PMC6782504

[B39] Yang Y. Liu S. Chowdhury S. A. DeAngelis G. C. Angelaki D. E. (2011). Binocular disparity tuning and visual-vestibular congruency of multisensory neurons in macaque parietal cortex. J. Neurosci. 31, 17905–17916 10.1523/JNEUROSCI.4032-11.2011 22159105 PMC3260792

[B40] Zhang T. Britten K. H. (2004). Clustering of selectivity for optic flow in the ventral intraparietal area. Neuroreport 15, 1941–1945 15305142 10.1097/00001756-200408260-00022

[B41] Zhang T. Heuer H. W. Britten K. H. (2004). Parietal area VIP neuronal responses to heading stimuli are encoded in head-centered coordinates. Neuron 42, 993–1001 10.1016/j.neuron.2004.06.008 15207243

